# Defected photonic crystal as propylene glycol THz sensor using parity-time symmetry

**DOI:** 10.1038/s41598-024-73477-7

**Published:** 2024-10-05

**Authors:** Zaky A. Zaky, M. Al-Dossari, V. D. Zhaketov, Arafa H. Aly

**Affiliations:** 1 Physics Department, Faculty of Science, TH-PPM Group, Beni-Suef University, Beni Suef, 62514 Egypt; 2 Academy of Scientific Research and Technology (ASRT), Cairo, Egypt; 3 Frank Laboratory of Neutron Physics, Joint Institute for Nuclear Research, Dubna, 141980 Russia; 4 Department of Physics, Faculty of Science, King Khalid University, Abha, 62529 Saudi Arabia; 5 Moscow Institute of Physics and Technology (State University), Dolgoprudnyi, Moscow oblast Russia; 6 Department of Technical Sciences, Western Caspian University, Baku, 1001, Azerbaijan

**Keywords:** Parity time symmetry, Propylene glycol, Refractive index, Defective photonic crystal, Optical materials, Computational science, Sensors and biosensors, Biophotonics, Photonic crystals

## Abstract

Detecting unsafe levels of chemical gases and vapors is essential in improving and maintaining a healthy environment for all to enjoy. Propylene glycol is a colorless, synthetic gas commonly used in medications, fragrances, and cosmetics. It causes side effects such as headaches, lightheadedness, nausea, and fainting. So, monitoring of propylene glycol is critically vital. This study uses a defected photonic crystal as a propylene glycol THz sensor. Due to the high absorption of propylene glycol, the intensity of the resonant confined mode is very small. As a result, the performance of the designed sensor seems unsatisfactory. We will use parity-time symmetry for the first time in THz to magnify the resonant confined mode to detect propylene glycol. The effect of microcavity thickness, incident angle, and gain/loss factor will be studied. The optimized sensor recorded distinguished results compared to other studies for the detection of propylene glycol.

**Supplementary Information:**

The online version contains supplementary material available at 10.1038/s41598-024-73477-7.

##  Introduction

Many hazardous and toxic chemical gases and vapors have an important impact in a variety of fields, including national defense, manufacturing in industries, medicine, and sustainability^1–4^. Propylene glycol (PG) is a chemical gas present in medications, fragrances, and cosmetics. PG is a great solvent for a variety of organic chemicals. PG is utilized as a surfactant or solvent for numerous items and as an active component in enamels, brakes, varnishes, paints, antifreeze, and engine coolants. PG significantly affects human organs (e.g., kidneys, eyes, liver, and skin)^5^. Headaches, lightheadedness, nausea, and fainting can all be brought on by PG^1,5^. PG is a flammable liquid that has the potential to catch fire. So, detection of PG is extremely important.

One-dimensional photonic crystals (1DPCs) are periodic optical structures that manipulate the transmittance, absorbance, and reflectance of photons in a similar way that periodic potentials in a semiconductor crystal affect electrons^6–9^. 1DPCs have unique properties that make them suitable for various applications in optics and photonics. 1DPC structure consists of alternating layers of different dielectric materials with varying refractive indices (RIs) arranged in a single dimension. Due to destructive interference from the periodic structure, a photonic band gap (PBG) is created and blocks the propagation of photons through it^10^. When light encounters the 1DPC, constructive and destructive interference patterns are created due to the differences in refractive indices. At certain frequencies, destructive interference dominates, resulting in a PBG where light cannot propagate through the 1DPC. The ability of 1DPC to control the propagation of light through PBGs is fundamental to their functionality in devices like filters, reflectors, and sensors^11–16^.

Nowadays, PT symmetry in optical periodic structures has emerged as a fascinating field of study for both technology and science since the publication of a collection of studies pointed out that the concepts of PT symmetry can be explored in a rich environment in photonics and optics^17–19^. The complex RI $\:n\left(x\right)$ in the optical media functions as complex potential $\:V\left(x\right)$. $\:{n}_{R}\left(x\right)$ denotes the real part of RI (RI distribution), and $\:{n}_{I}\left(x\right)$ represents the loss and gain profiles of the medium. PT symmetry indicates that $\:{n}_{R}$ must be an even function, and the function of gain-loss ($\:{n}_{I}$) must be an odd as follows: $\:{n}_{R}\left(x\right)={n}_{R}(-x)$ and $\:{n}_{I}\left(x\right)=-{n}_{I}(-x)$. Fortunately, all of these ingredients, loss, gain, and refractive index, are easily implemented in PCs. The confined peak will be magnified by achieving the PT symmetry conditions^20^.

In this study, an easy, rapid, efficient, and sensitive model of a PG sensor in the THz range using 1DPC with magnified confined resonance. Taking the absorption of the PG analyte into account makes our results very close to the experiment. The absorption of the PG analyte causes a decrease in the intensity of the confined peaks and the performance of the model. We used PT symmetry to magnify the peaks and enhance the performance to overcome this issue.

## Theoretical model and basic equations

**Fig. 1:**
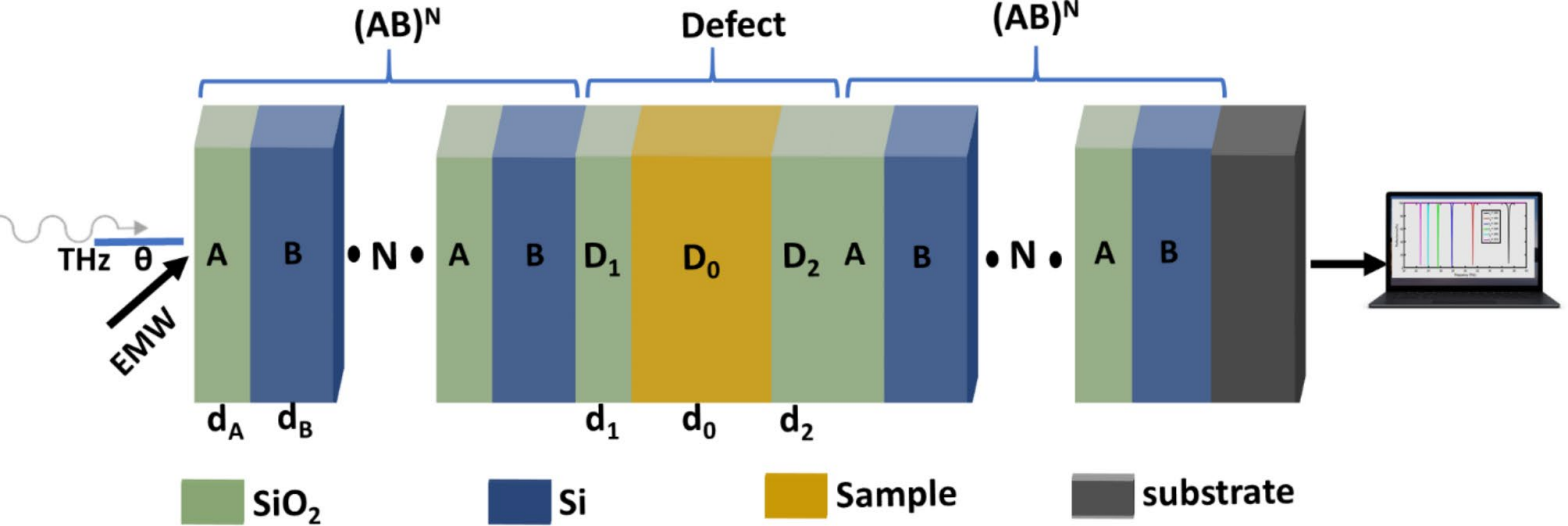
Schematic of $\:{\left(AB\right)}^{N}\left({D}_{1}{D}_{0}{D}_{2}\right){\left(AB\right)}^{N}*Si{O}_{2}\:substrate$ structure as PG THz sensor.

The suggested PG THz sensor is composed of a 1DPC using $\:Si{O}_{2}$ (A) and $\:Si$ (B) layers, as clear in Fig. 1. The periodic 1DPC contains a defect sample ($\:{D}_{0}$) layer sandwiched between two $\:Si{O}_{2}$ layers ($\:{D}_{1}$ and $\:{D}_{2}$) to prevent $\:Si$ oxidation. The whole suggested PG THz sensor is represented as $\:{\left(AB\right)}^{N}\left({D}_{1}{D}_{0}{D}_{2}\right){\left(AB\right)}^{N}*Si{O}_{2}\:substrate$. $\:N=5$ is the number of $\:Si{O}_{2}*Si$ periods. The sample microcavity (SMC) will be occupied with an aqueous solution with different concentrations of PG.

In the THz region, the RI and amplitude absorption ($\:\alpha\:$) of PG aqueous solution with different concentrations of PG were measured over a frequency range from 0.30 THz to 2.50 THz^21^. By fitting the RI and amplitude absorption of aqueous solution with different concentrations of PG from 0% (water) to 100% (PG), we get the following equations as a function of frequency ($\:f$) from 0.5 THz to 2.5 THz:

For 0% PG, 0 mol/l (water):

1 $$\:n=-\:0.07042\:{f}^{3}\:+\:0.4293{\:f}^{2}\:-\:0.9245\:f\:+\:2.744\:\:\:\:\:\:\:\left({R}^{2}=0.994\right),$$

2 $$\:\alpha\:=-17.0559{\:f}^{2}\:+\:151.258.f\:+\:81.2348\:\:\:\:\:({R}^{2}=0.998)$$

For 20% PG, 2.73 mol/l:

3 $$\:n=-\:0.02815\:{f}^{3}\:+\:0.2126{\:f}^{2}\:-\:0.5611\:f+\:2.400\:\:\:\:\:\:\:\left({R}^{2}=0.995\right),$$

$$\:\alpha\:=-\:7.85127{\:f}^{2}\:+\:73.2429\:f\:+\:38.009\:\:\:\:\:\left({R}^{2}=0.996\right)$$ (4)

For 40% PG, 5.47 mol/l:

5 $$\:n=-\:0.01924\:{f}^{3}\:+\:0.1592{\:f}^{2}\:-\:0.4537\:f+\:2.278\:\:\:\:\:\:\:\left({R}^{2}=0.998\right),$$

6 $$\:\alpha\:=-\:0.744781{\:f}^{2}\:+\:59.901\:f\:+\:13.0406\:\:\:\:({R}^{2}=0.999)$$

For 60% PG, 8.20 mol/l:

7 $$\:n=-\:0.005979\:{f}^{3}\:+\:0.06884{\:f}^{2}\:-\:0.2628\:\:f+\:2.054\:\:\:\:\:\:\:\left({R}^{2}=0.997\right),$$

8 $$\:\alpha\:=-\:2.78338{\:f}^{2}\:+\:55.3389\:f\:+\:4.1937\:\:({R}^{2}=0.999)$$

For 80% PG, 10.93 mol/l:

9 $$\:n=-\:0.003407\:{f}^{3}\:+\:0.03876{\:f}^{2}\:-\:0.1872\:f+\:1.913\:\:\:\:\:\:\left({R}^{2}=0.998\right),$$

$$\:\alpha\:=-\:0.830281{\:f}^{2}\:+\:41.9095\:f\:+\:5.00986\:\:\left({R}^{2}=0.999\right)$$ (10)

For 100% PG, 13.67 mol/l:

11 $$\:n=0.01757\:{f}^{3}\:-\:0.05735{\:f}^{2}\:-\:0.05439\:f+\:1.723\:\:\:\:\:\:\:\left({R}^{2}=0.997\right),$$

12 $$\:\alpha\:=-\:3.75508{\:f}^{2}\:+\:42.1828\:f\:-\:8.95137\:\:\:\:({R}^{2}=0.998)$$

From these RIs and amplitude absorption relations, the complex RI of aqueous solution with different concentrations of PG from 0% (water) to 100% (PG) is calculated as follows:

13 $$\:\stackrel{-}{n}=n+i\frac{{C}_{0}}{4\pi\:f}\:\alpha\:,$$

where $\:{C}_{0}$ is the vacuum’s speed of light. The RIs and thicknesses of $\:A$ and $\:B$ layers are 2.1, 3.4, 30 μm, and 7 μm, respectively. The thickness of the layers $\:{D}_{1}$ and $\:{D}_{2}$ is 2 μm.

The transmittance (T) of the TE polarized THz wave (Transverse Electric) due to the interaction with the PG sensor will be investigated with the transfer matrix method (TMM) as follows^22–27^:

The total matrix of the structure is:

14 $$\:\text{A}=\left|\begin{array}{cc}{A}_{11}&\:{A}_{12}\\\:{A}_{21}&\:{A}_{22}\end{array}\right|={\left({a}_{A}{a}_{B}\right)}^{N}\left({a}_{D1}{a}_{D0}{a}_{D2}\right){\left({a}_{A}{a}_{B}\right)}^{N},$$

where $$\:{A}_{11}$$, $$\:{A}_{12},$$$$\:{A}_{21}$$, and $$\:{A}_{22}$$ are elements of the total matrix. The matrix of each layer can be represented as follows:

15 $$\:{a}_{A}=\left[\begin{array}{cc}cos{\sigma\:}_{A}&\:\left(-\frac{i}{{\varnothing\:}_{A}}\right)sin{\sigma\:}_{A}\\\:-i{\varnothing\:}_{A}sin{\sigma\:}_{A}&\:cos{\sigma\:}_{A}\end{array}\right],$$

16 $$\:{a}_{B}=\left[\begin{array}{cc}cos{\sigma\:}_{B}&\:\left(-\frac{i}{{\varnothing\:}_{B}}\right)sin{\sigma\:}_{B}\\\:-i{\varnothing\:}_{B}sin{\sigma\:}_{B}&\:cos{\sigma\:}_{B}\end{array}\right],$$

17 $$\:{a}_{D1}=\left[\begin{array}{cc}cos{\sigma\:}_{D1}&\:\left(-\frac{i}{{\varnothing\:}_{D1}}\right)sin{\sigma\:}_{D1}\\\:-i{\varnothing\:}_{D1}sin{\sigma\:}_{D1}&\:cos{\sigma\:}_{D1}\end{array}\right],$$

18 $$\:{a}_{D0}=\left[\begin{array}{cc}cos{\sigma\:}_{D0}&\:\left(-\frac{i}{{\varnothing\:}_{D0}}\right)sin{\sigma\:}_{D0}\\\:-i{\varnothing\:}_{D0}sin{\sigma\:}_{D0}&\:cos{\sigma\:}_{D0}\end{array}\right],$$

19 $$\:{a}_{D2}=\left[\begin{array}{cc}cos{\sigma\:}_{D2}&\:\left(-\frac{i}{{\varnothing\:}_{D2}}\right)sin{\sigma\:}_{D2}\\\:-i{\varnothing\:}_{D2}sin{\sigma\:}_{D2}&\:cos{\sigma\:}_{D2}\end{array}\right],$$

where $\:{n}_{i}$ and $\:{\theta\:}_{i}$ are the RI and incident angle.

20 $$\:{\sigma\:}_{i}=\frac{2\pi\:}{\lambda\:}{d}_{i}{n}_{i}cos{\theta\:}_{i}\:$$

21 $$\:{\varnothing\:}_{i}={n}_{i}\:cos\left({\theta\:}_{i}\right),\:i=\text{A},\:\text{B},\:{D}_{1},\:{D}_{0},\:\text{a}\text{n}\text{d}\:{D}_{2}$$

The angles of incidence for each layer are calculated using Snell’s law as follows:

22 $$\:{n}_{0}\:sin\left({\theta\:}_{0}\right)={n}_{A}\:sin\left({\theta\:}_{A}\right)={n}_{B}\:sin\left({\theta\:}_{B}\right)={n}_{D1}\:sin\left({\theta\:}_{D1}\right)={n}_{D0}\:sin\left({\theta\:}_{D0}\right)={n}_{D2}\:sin\left({\theta\:}_{D2}\right)={n}_{s}\:sin\left({\theta\:}_{s}\right)$$

To calculate $\:\left({a}_{A}{a}_{B}\right)$ for N periods, the Chebyshev polynomials of the second kind are used^28^. The transmittance coefficient is given by:

23 $$\:t=\frac{2{\varnothing\:}_{0}}{\left({A}_{11}+{A}_{12}{\varnothing\:}_{s}\right){\varnothing\:}_{0}+\left({A}_{21}+{A}_{22}{\varnothing\:}_{s}\right)},$$

where $\:{\varnothing\:}_{0}$ and $\:{\varnothing\:}_{s}$ are for ambient medium and $\:Si{O}_{2}$ substrate. The total transmittance of the $\:{\left(AB\right)}^{N}\left({D}_{1}{D}_{0}{D}_{2}\right){\left(AB\right)}^{N}*Si{O}_{2}\:substrate$ structure is given by:

24 $$\:T\left(\%\right)=100\times\:\frac{{p}_{s}}{{p}_{0}}{⌊t⌋}^{2},$$

Besides, Bloch’s theorem is used to ensure the region of photonic bandgap (PBG) of the discussed THz PG sensor structure (infinite periods) as follows^29^:

25 $$\:\text{cos}\left(Kd\right)=\text{cos}\left({k}_{A}{d}_{A}\right)\text{cos}\left({k}_{B}{d}_{B}\right)-\frac{1}{2}\left(\frac{{p}_{B}}{{p}_{A}}+\frac{{p}_{A}}{{p}_{B}}\right)\text{sin}\left({k}_{A}{d}_{A}\right)\text{sin}\left({k}_{B}{d}_{B}\right).$$

where $\:K$ is the z component of the Bloch wave vector.

TMM has been used to simulate filters^30^, sensors^31–35^, etc. Wang et al.^36^ experimentally and theoretically (using TMM) studied the reflectance of 1DPC as a reflector. The experiment coincided with the results using TMM. Gutierrez et al.^37^ fabricated and modeled a device to sense any change in chemical reaction, optical path, refractive index, temperature, and roughness while forming porous films. In 2022, Zhang et al.^38^ experimentally and theoretically (using TMM) designed an optical filter using PT symmetry. These numerical and experimental results recorded very good matches with accepted discrepancy because of the non-optimized design of the coupling coefficients and fabrication errors.

## Results and discussions

**Fig. 2:**
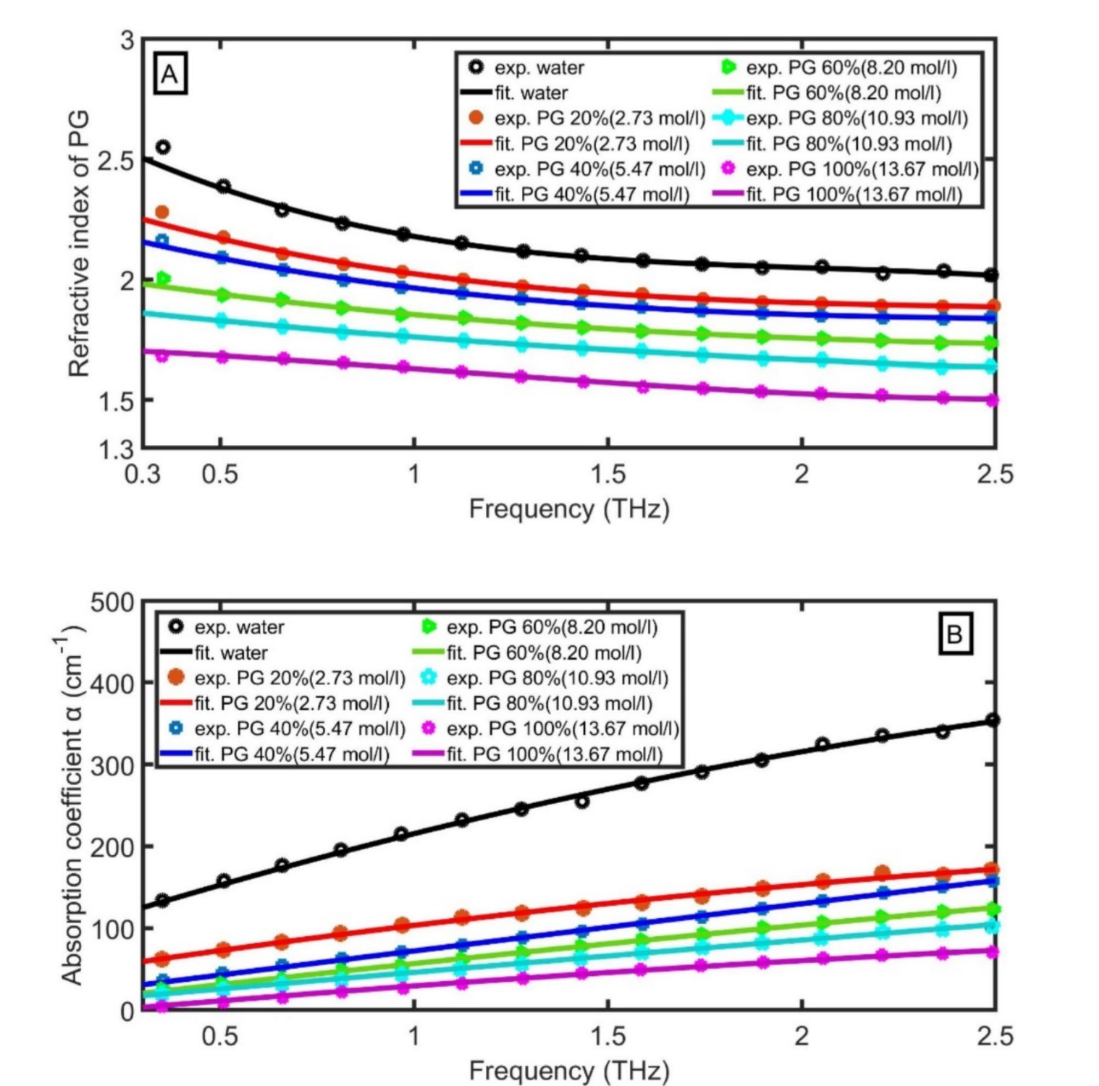
Measured and fitted (**A**) RI and (**B**) amplitude absorption of aqueous solution with different concentrations of PG from 0% (water) to 100% (PG).

Figure 2A, B clear the RI and amplitude absorption of aqueous solution with different concentrations of PG from 0% (water) to 100% (PG). By increasing the PG concentration, the RI and amplitude absorption decrease. Besides, the RIs of PG aqueous solutions decrease with the frequency increase, but the amplitude absorption increases with the frequency increase.

**Fig. 3:**
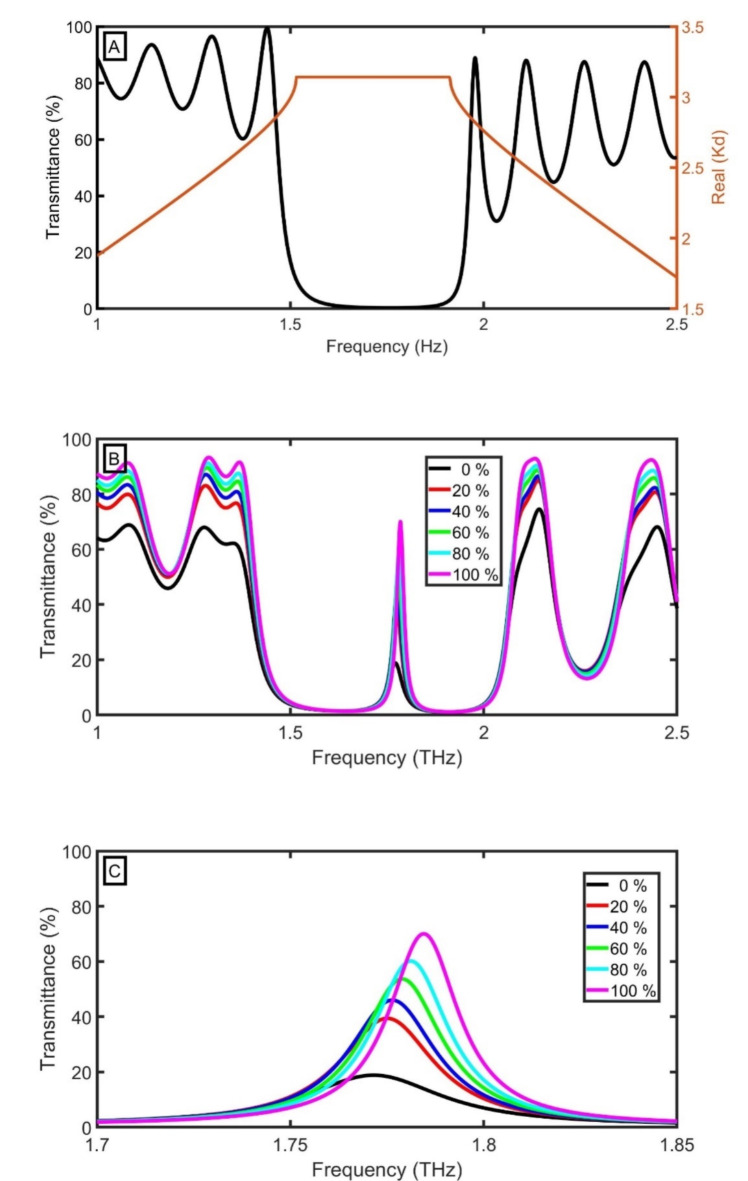
Transmittance of discussed THz PG sensor at different concentrations of PG from 0% (water) to 100% (PG): (**A**) without defect, (**B**) with defect, and (**C**) peaks shift.

By modeling a 1DPC made of layers A and B ($\:Si{O}_{2}$ and $\:Si$) without defect layers $\:\left({D}_{1}{D}_{0}{D}_{2}\right)$ using TMM and Bloch’s theorem (Fig. 3A), a photonic bandgap (PBG) extended from 1.51551 THz to 1.91286 THz as a result of the RI contrast between $\:Si{O}_{2}$ and $\:Si$. The position of the PBG is compatible with the following relation (for normal incidence)^39^:

26 $$\:{f}_{PBG}=\frac{{c}_{0}\:({n}_{A}+{n}_{B})}{4({d}_{A}+{d}_{B})\left({n}_{A}{n}_{B}\right)}.$$

Accourding to Eq. (26), $\:{f}_{PBG}$=1.56 THz. In the case of the defect $\:{\left(AB\right)}^{N}\left({D}_{1}{D}_{0}{D}_{2}\right){\left(AB\right)}^{N}\text{*}substrate$, the thickness of $\:{D}_{0}$ is 12 μm. The sample cavity at the center of the defect will be occupied with an aqueous solution with different concentrations of PG from 0% (water) to 100% (PG). Due to the breaking of the periodicity with defect layers $\:\left({D}_{1}{D}_{0}{D}_{2}\right)$, a confined peak appeared inside the PBG, as clear in Fig. 3B. In Fig. 3C, by increasing concentrations of PG from 0% (water) to 100% (pure PG), the confined peak is shifted to blue THz waves from 1.77154 THz (for water) to 1.78461 THz (for pure PG) due to the increase of RI, according to the standing wave equation^39^:

27 $$\:\frac{m\:{C}_{0}}{{f}_{R}}=2{n}_{sample}\:{D}_{defect},$$

where m is the order. With increasing the concentrations of PG, the RI of PG ($\:{n}_{sample}$) decreases, the effective RI ($\:{n}_{eff}$) decreases, and the $\:{f}_{R}$ increases.

The efficacy of any sensor is often measured by different parameters such as sensitivity (S), bandwidth (FWHM), figure of merit (FoM), quality factor (Q), and detection limit (DL) as follows:

28 $$\:S\:=\frac{\varDelta\:{f}_{R}}{\varDelta\:{n}_{sample}},$$

29 $$\:FoM=\frac{S}{FWHM},$$

30 $$\:\begin{array}{l}Q=\frac{{f}_{R}}{FWHM},\end{array}$$

31 $$\:DL=\frac{{f}_{R}}{20\:S\:Q-factor},$$

Table 1 shows the different parameters, such as sensitivity (S), bandwidth (FWHM), figure of merit (FoM), Q-factor, and detection limit (DL) of the described THz sensor at different concentrations of PG. By increasing the concentrations of PG, the $\:{f}_{R}$ increases, the intensity of confined peak increases, the FWHM decreases, the S changes between 0.6 GHz/mol/l and 1.3 GHz/mol/l, the FoM changes between 0.02 l/mol and 0.06 l/mol, the Q-factor increases from 43.42 to 83.39, and the DL changes between 0.83 mol/l to 2.23 mol/l. In general, the efficiency of the sensor needs to be enhanced. So, different geometrical will be optimized to increase the performance.

**Table 1:** The $\:{f}_{R}$, Intensity, FWHM, S, FoM, Q-factor, and DL of the designed sensor versus concentration (Conc.) Of PG.

Conc. (%)	Conc. (mol/l)	$\:{f}_{R}$ (THz)	Intensity (%)	FWHM (THz)	S (GHz/mol/l)	FoM (l/mol)	Q-factor	DL (mol/l)
0	0	1.77154	18.9	0.041	–	–	43.42	–
20	2.73	1.77516	39.4	0.029	1.3	0.05	61.42	1.09
40	5.47	1.77681	46.0	0.027	0.6	0.02	66.30	2.23
60	8.2	1.77894	53.7	0.025	0. 9	0.03	72.02	1.58
80	10.93	1.78108	69.2	0.023	0. 8	0.03	76.77	1.48
100	13.67	1.78461	70.0	0.021	1.3	0.06	83.39	0.83

**Fig. 4:**
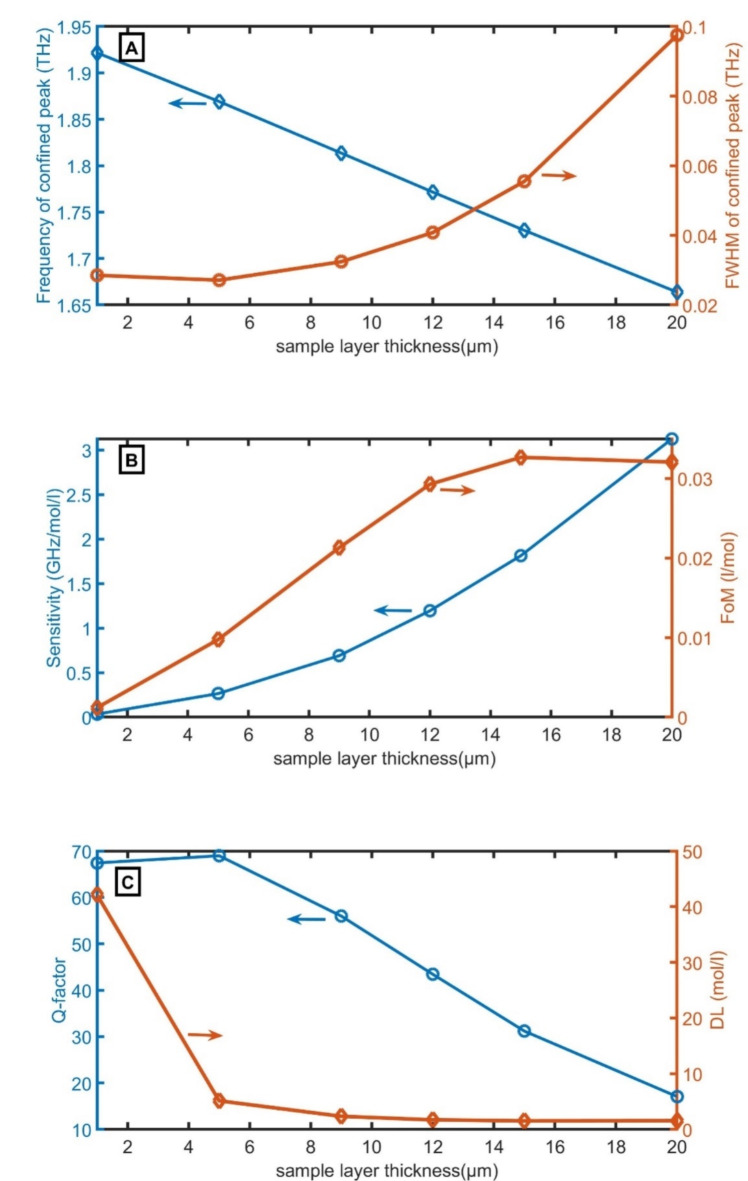
performance parameters of the designed sensor; (**A**) $\:{f}_{R}$ and FWHM, (**B**) S and FoM, and (**C**) Q-factor and DL versus the sample cavity width.

The impact of sample cavity thickness ($\:{D}_{0}$) on the designed sensor will be investigated as clear in Fig. 4A–C. The $\:{f}_{R}$, FWHM, S, FoM, Q-factor, and DL of the designed sensor are tuned by changing the sample cavity thickness. By increasing the width of $\:{D}_{0}$, the frequency of the confined peak decreases according to Eq. (27). On the other hand, the FWHM increases from 0.03 THz to 0.10 THz, the S increases from 0.03 GHz/mol/l to 3.13 GHz/mol/l with increasing the width of $\:{D}_{0}$ from 1 μm to 20 μm. At the narrow cavity width (1–15 μm), the moving FoM is very high. The FoM at 1 μm is 0.001 l/mol, at 5 μm is 0.010 l/mol, at 9 μm is 0.021 l/mol, at 12 μm is 0.029 l/mol, and at 15 μm is 0.033 l/mol. At cavity width wider than 15 μm (20 μm), the FoM slightly decreases (0.032 l/mol). Both Q-factor and DL decrease with increasing the width of $\:{D}_{0}$ from 1 μm to 20 μm to record the lowest value at 20 μm. As the width of $\:{D}_{0}$ of 15 μm has the best FoM than at 20 μm, the width of $\:{D}_{0}$ of 15 μm will be selected for the following optimization.

**Fig. 5:**
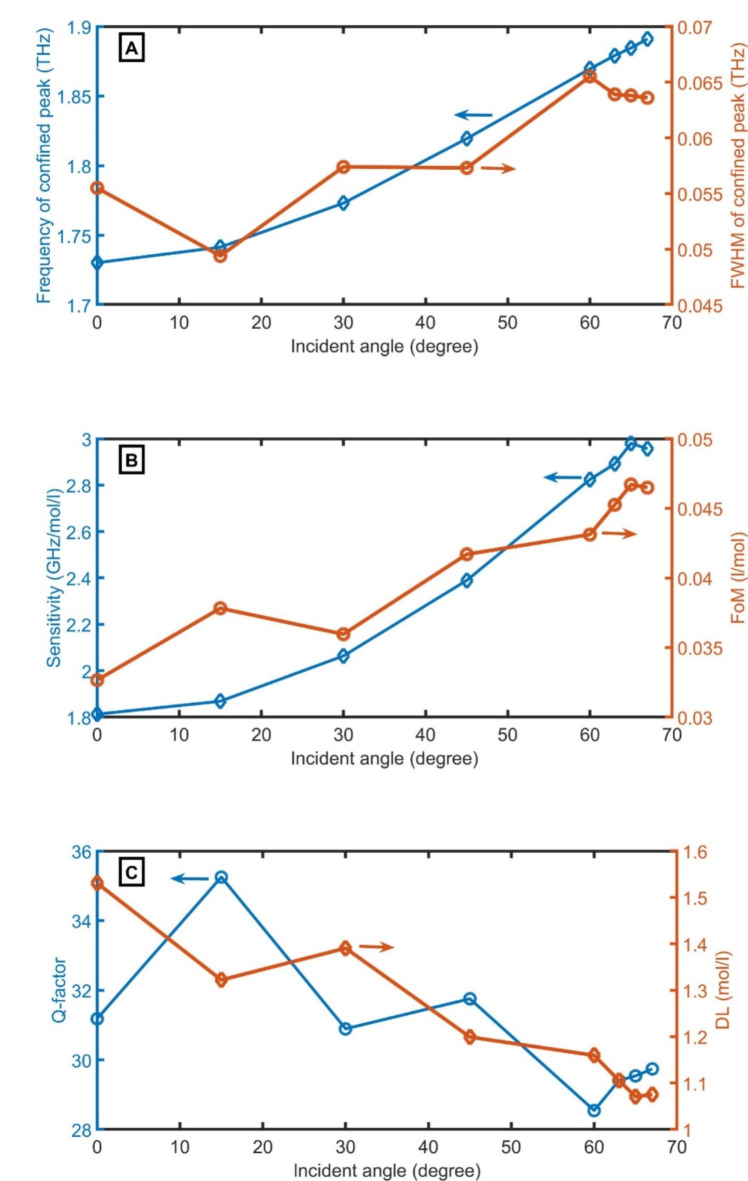
performance parameters of the designed sensor; (**A**) $\:{f}_{R}$ and FWHM, (**B**) S and FoM, and (**C**) Q-factor and DL versus the angle of incidence.

The influence of angle of incidence ($\:\theta\:$) on the designed sensor will be studied as clear in Fig. 5A–C. The $\:{f}_{R}$, FWHM, S, FoM, Q-factor, and DL of the designed sensor are varied by moving the $\:\theta\:$. By increasing $\:\theta\:$, the frequency of the confinedpeak increases according to Brag Snell’s law^40^. On the other hand, the FWHM vibrates from 0.049 THz to 0.065 THz, the S increases from 1.81 GHz/mol/l to 2.96 GHz/mol/l, the FoM vibrates from 0.03 l/mol to 0.05 l/mol, with increasing the θ from 0 degrees to 67 degrees. Both Q-factor and DL decrease with increasing the θ from 0 degrees to 67 degrees. At higher angles than 67 degree, the confined peak diapeared. The θ of 67 degrees in principle, will be selected for the following optimization.

**Fig. 6:**
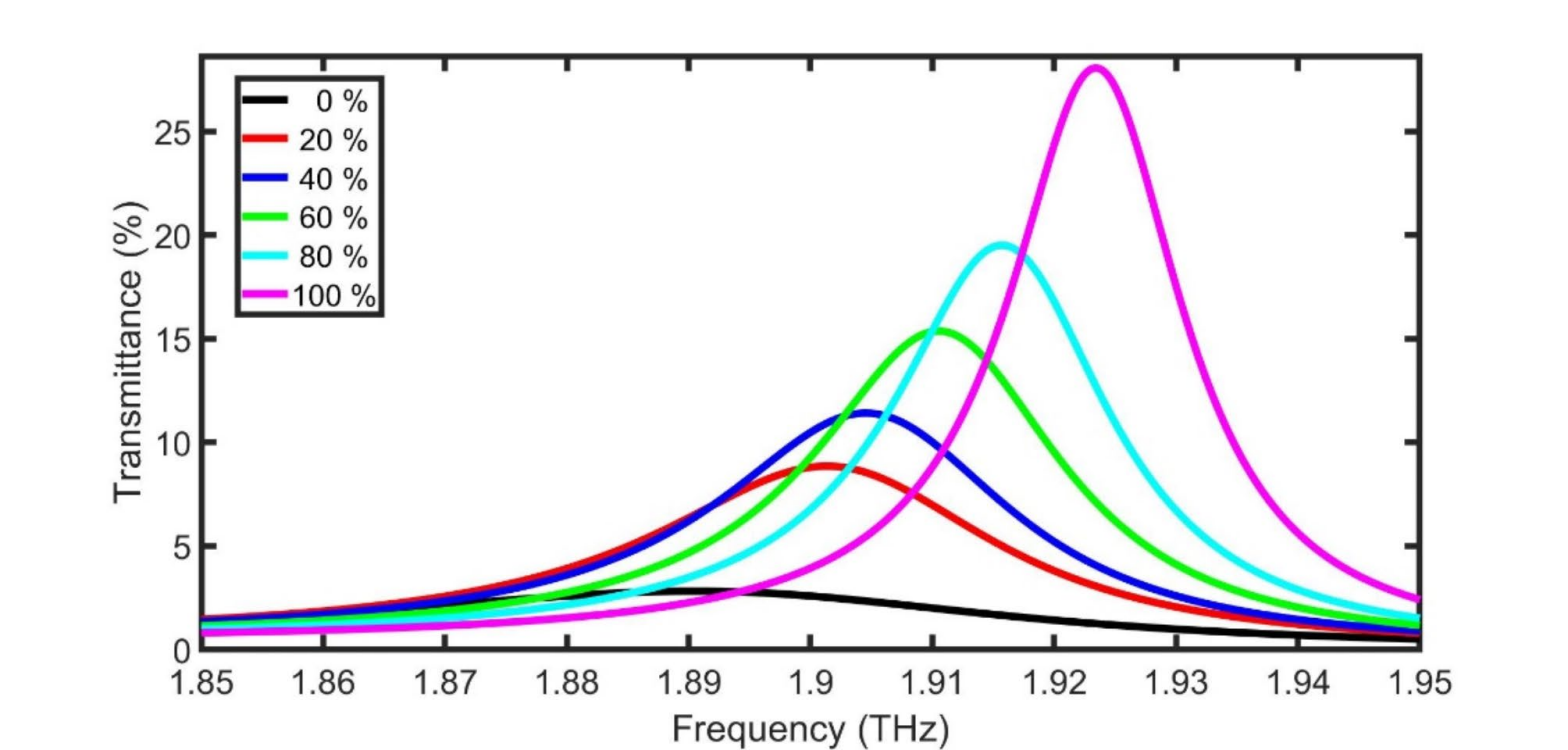
Transmittance of discussed THz PG sensor for different concentrations of PG from 0% (water) to 100% (PG) at $\:{D}_{0}$ of 15 μm and θ of 67 degrees.

Figure 6 clears the variation of transmittance of discussed THz PG sensor for different concentrations of PG at $\:{D}_{0}$ of 15 μm and θ of 67. in spite of the confinedpeak shift is enhanced after the optimization comparing with Fig. 3C, the FWHM and intensity of peak should be enhanced. To solve this issue, the confinedpeaks need to be magnified using the well known parity-time (PT) symmetric 1DPC (PT-1DPC)^41–44^.

**Fig. 7:**
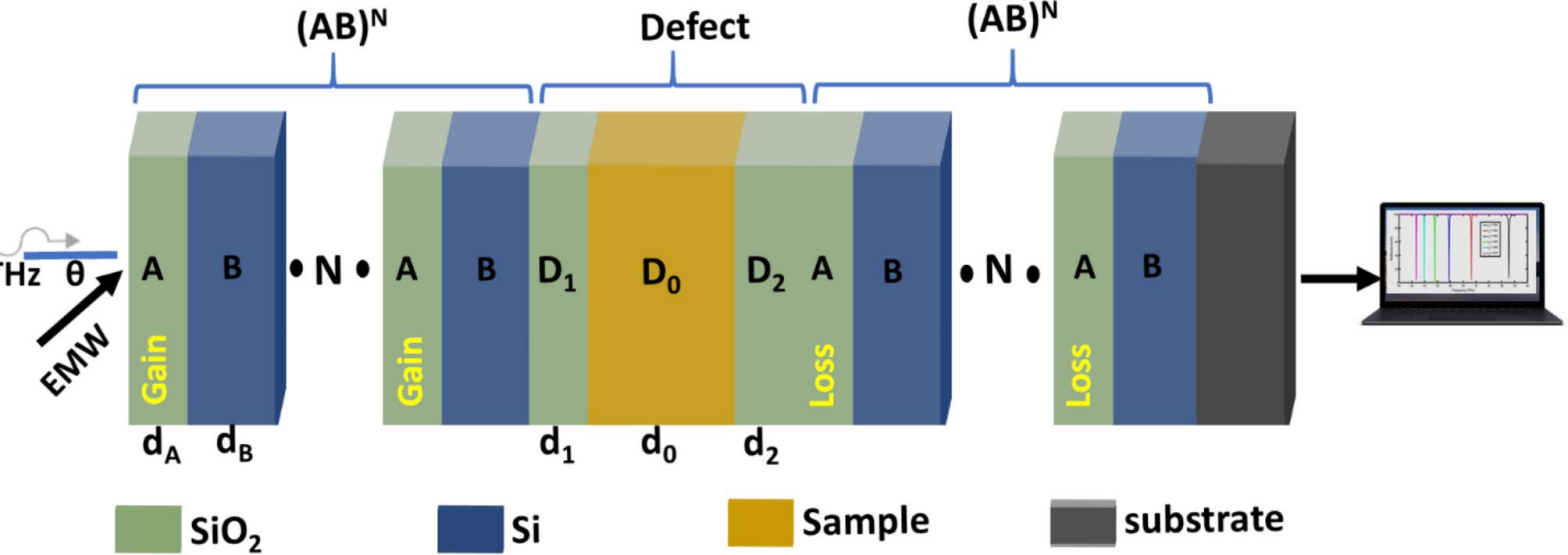
Schematic of $\:{\left(AB\right)}^{N}\left({D}_{1}{D}_{0}{D}_{2}\right){\left(AB\right)}^{N}*Si{O}_{2}\:substrate$ structure as PG THz sensor after PT symmetry modifications.

By doping the $\:Si{O}_{2}$ layer with quantum dots, the gain and loss layer can be prepared^20,45^. The gain layer will be externally pumped with an optical pump^46^. The gain layer will be excited by the optical pump, and a certain frequency of photons will be absorbed by the quantum dots. Quantum dots re-emit these photons with the same frequency of propagated distinct peak by stimulated emission. The coupling between distinct peaks and re-emitted frequencies causes the magnification process. The complex RI of gain and loss layers are $\:{n}_{gain}={n}_{R}-0.01\:Q$ and $\:{n}_{loss}={n}_{R}+0.01\:Q$, where $\:Q$ is the gain/loss factor. Figure 7 clears the schematic of PG sensor after taking the PT conditions into account.

**Table 2:** The $\:{f}_{R}$ of the designed sensor versus Q.

Q	$\:{f}_{R}\:$(THz)	Q	$\:{f}_{R}\:$(THz)	Q	$\:{f}_{R}\:$(THz)
0	1.4	10	27.5	12.8	905
1	1.6	11	77	12.9	572
2	1.9	12	878	13	393
3	2.3	12.1	1534	14	52
4	2.8	12.2	3339	15	18
5	3.5	12.3	12,482	16	9
6	4.5	**12.4**	**2,798,600**	17	7
7	6	12.5	16,390	18	5
8	9	12.6	3792	19	3
9	14	12.7	1636	20	1

**Fig. 8:**
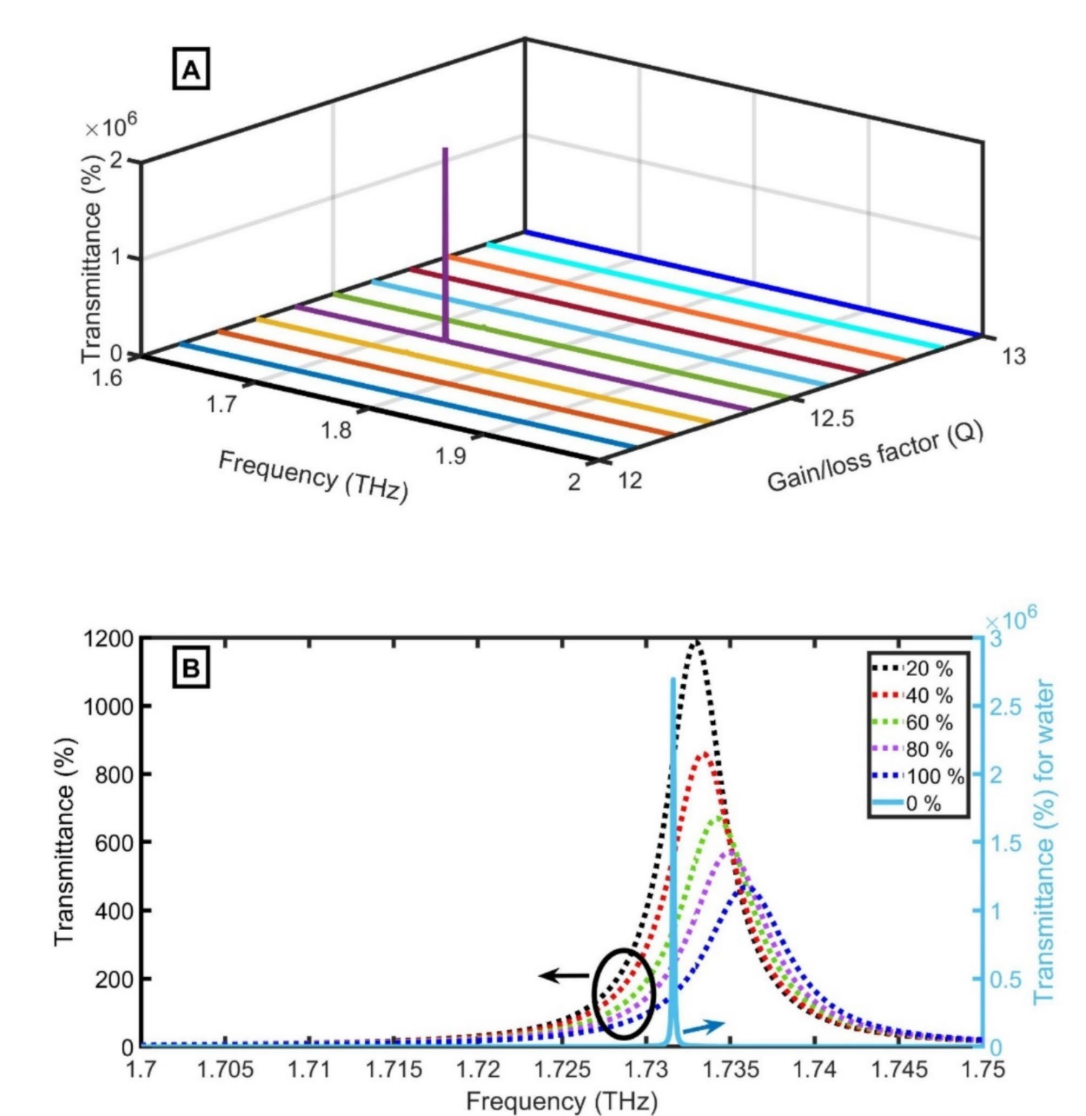
Transmittance of discussed THz PG sensor (**A**) for 0% concentration of PG at different gain/loss factor (Q), (**B**) for Q of 12.4 at different PG concentrations.

Using PT symmetry conditions in this study affects the position of the bandgap due to the change in the complex refractive index, as clear in supplementary data. Now, the transmittance of discussed THz PG sensor for 0% concentration of PG is checked. The structure recorded low transmittance again at $\:Q=1$. So, the transmittance is investigated at different Q. As clear in Table 2, the transmittance gradually increases with increasing the value of Q from 0 to 12.4. Then, the transmittance decreases. As clear in Fig. 8A, the transmittance of the discussed THz PG sensor has the highest value of $\:3.8\times\:{10}^{6}$ % at Q of 12.4. Figure 8B clears the variation of transmittance of discussed THz PG sensor for different concentrations of PG at $\:{D}_{0}$ of 15 μm and θ of 67 after achieving the PT symmetry conditions. The magnification (transmittance) of the confined peak at 0% PG concentration has the highest value ($\:3.8\times\:{10}^{6}$ %). By increasing the PG concentration from 20 to 40%, 60%, 80% and 100%, the transmittance of the confined peak changed from 1185 to 858%, 670%, 569%, to 468%. Due to the drop in transmittance with increasing PG concentration, the FWHM increases. From results in Fig. 8B, it can be concluded that the investigated structure is very suitable for the detection of low concentrations close to 0% than higher concentrations. Table 3 ensures that the described sensor has high sensitivity compared to others. As fast as the pulse lasts, the optical response of the proposed sensor happens instantly^47^ which is in order of several nanoseconds^48^.

**Table 3:** Comparison study with other devices.

References	S (GHz / RIU)	Materials
2014^49^	2.998	+ve and -ve refractive index materials
2016^50^	2	Matrix of photonic molecules
2019^51^	12.78	Array of microstructured holes in Al
2020^52^	0.95	Enhanced toroidal localized spoof surface plasmons
2021^53^	0.496	Electric Permittivity sensor using Metamaterial
2021^54^	0.78	Toroidal Metasurface
2022^32^	15.14	1D-PC using Magnetized Cold Plasma
This work	62.6	1D-PC using PT symmetry

## Conclusion

In this study, magnified confined peak of defective PC using PT symmetry as a PG sensor in the THz region has been discussed. The optimized sensor recorded a sensitivity of 62.6 GH/RIU. The confined peak is strongly magnified at lower concentrations ($\:3.8\times\:{10}^{6}$ % at 0% concentration) than at higher concentrations. So, the investigated structure is very suitable for the detection of low concentrations close to 0% with high sensitivity compared to others. Finally, PT symmetry can be considered as an excellent solution for high absorption of analytes.

## Electronic supplementary material

Below is the link to the electronic supplementary material.

Supplementary Material 1

## Data Availability

Requests for materials or code should be addressed to Zaky A. Zaky.

## References

[1] Adamyan, Z. et al. Nanocomposite sensors of propylene glycol, dimethylformamide and formaldehyde vapors. *J. Sens. Sens. Syst.* **7**, 31–41. 10.5194/jsss-7-31-2018 (2018).

[2] Zaky, Z. A., Al-Dossari, M., Hendy, A. S., Badawy, W. M. & Aly, A. H. Periodic open and closed resonators as a biosensor using two computational methods. *Sci. Rep.* **14**, 11943. 10.1038/s41598-024-61987-3 (2024). 38789449 10.1038/s41598-024-61987-3 PMC11126621

[3] Zaky, Z. A., Al-Dossari, M., Sharma, A., Hendy, A. S. & Aly, A. H. Theoretical optimisation of a novel gas sensor using periodically closed resonators. *Sci. Rep.* **14**, 2462. 10.1038/s41598-024-52851-5 (2024). 38291144 10.1038/s41598-024-52851-5 PMC10828414

[4] Zaky, Z. A., Mohaseb, M. & Aly, A. H. Detection of hazardous greenhouse gases and chemicals with topological edge state using periodically arranged cross-sections. *Phys. Scr.* **98**, 065002. 10.1088/1402-4896/accedc (2023).

[5] Robertson, O. et al. Tests for the chronic toxicity of propylexe glycol and triethylene glycol on monkeys and rats by vapor inhalation and oral administration. *J. Pharmacol. Exp. Ther.* **91**, 52–76 (1947). 20265820

[6] Han, P. & Wang, H. Extension of omnidirectional reflection range in one-dimensional photonic crystals with a staggered structure. *J. Opt. Soc. Am. B* **20**, 1996–2001. 10.1364/JOSAB.20.001996 (2003).

[7] Zaky, Z. A. et al. Theoretical optimization of Tamm Plasmon Polariton structure for pressure sensing applications. *Opt. Quant. Electron.* **55**, 738. 10.1007/s11082-023-05023-0 (2023).

[8] Hojo, H. & Mase, A. Dispersion relation of electromagnetic waves in one-dimensional plasma photonic crystals. *J. Plasma Fusion Res.* **80**, 89–90. 10.1585/jspf.80.89 (2004).

[9] Wu, F., Liu, T., Chen, M. & Xiao, S. Photonic bandgap engineering in hybrid one-dimensional photonic crystals containing all-dielectric elliptical metamaterials. *Opt. Express* **30**, 33911–33925. 10.1364/OE.469368 (2022). 36242416 10.1364/OE.469368

[10] Al-Dossari, M., Zaky, Z. A., Awasthi, S. K., Amer, H. A. & Aly, A. H. Detection of glucose concentrations in urine based on coupling of Tamm–Fano resonance in photonic crystals. *Opt. Quant. Electron.* **55**, 484. 10.1007/s11082-023-04621-2 (2023).

[11] Zaky, Z. A. et al. Theoretical analysis of porous silicon one-dimensional photonic crystal doped with magnetized cold plasma for hazardous gases sensing applications. *Opt. Quant. Electron.* **55**, 584. 10.1007/s11082-023-04907-5 (2023).

[12] Abohassan, K. M., Ashour, H. S. & Abadla, M. M. Tunable wide bandstop and narrow bandpass filters based on one-dimensional ternary photonic crystals comprising defects of silver nanoparticles in water. *J. Phys. Chem. Solids* **161**, 110484. 10.1016/j.jpcs.2021.110484 (2022).

[13] Zaky, Z. A., Al-Dossari, M., Hendy, A. S., Zayed, M. & Aly, A. H. Gamma radiation detector using Cantor quasi-periodic photonic crystal based on porous silicon doped with polymer. *Int. J. Mod. Phys. B*. 10.1142/S0217979224504095 (2024).

[14] Suthar, B. & Bhargava, A. Biosensor Application of One-Dimensional Photonic Crystal for Malaria Diagnosis, *Plasmonics* **16** 59–63. 10.1007/s11468-020-01259-8 (2021).

[15] Awasthi, S. Multichannel tunable polarizing filter properties of one-dimensional ternary photonic crystal containing single-negative materials. *Indian J. Phys.*. 10.1007/s12648-020-01939-5 (2021).

[16] Zaky, Z. A., Al-Dossari, M., Hendy, A. S. & Aly, A. H. Studying the impact of interface roughness on a layered photonic crystal as a sensor. *Phys. Scr.* **98**, 105527. 10.1088/1402-4896/acfa4a (2023).

[17] Guo, A. et al. Observation of PT-symmetry breaking in complex optical potentials. *Phys. Rev. Lett.* **103**, 093902. 10.1103/PhysRevLett.103.093902 (2009). 19792798 10.1103/PhysRevLett.103.093902

[18] Rüter, C. E. et al. Observation of parity–time symmetry in optics. *Nat. Phys.* **6**, 192–195. 10.1038/nphys1515 (2010).

[19] Ramezani, H., Kottos, T., El-Ganainy, R. & Christodoulides, D. N. Unidirectional nonlinear PT-symmetric optical structures. *Phys. Rev. A* **82**, 043803. 10.1103/PhysRevA.82.043803 (2010).

[20] Zaky, Z. A., Alamri, S., Zhaketov, V. & Aly, A. H. Refractive index sensor with magnified resonant signal. *Sci. Rep.* **12**, 13777. 10.1038/s41598-022-17676-0 (2022). 35961999 10.1038/s41598-022-17676-0 PMC9374705

[21] Musina, G. R. et al. Optimal hyperosmotic agents for tissue immersion optical clearing in terahertz biophotonics. *J. Biophotonics* **13**, e202000297. 10.1002/jbio.202000297 (2020). 32881362 10.1002/jbio.202000297

[22] Yeh, P. *Optical Waves in Layered Media* (Wiley New York, 1988).

[23] Zaky, Z. A., Mohaseb, M., Hendy, A. S. & Aly, A. H. Design of phononic crystal using open resonators as harmful gases sensor. *Sci. Rep.* **13**, 9346. 10.1038/s41598-023-36216-y (2023). 37291147 10.1038/s41598-023-36216-y PMC10250530

[24] Zaky, Z. A. et al. Photonic crystal with magnified resonant peak for biosensing applications. *Phys. Scr.* **98**, 055108. 10.1088/1402-4896/accbf1 (2023).

[25] Zaky, Z. A., Alamri, S., Zohny, E. I. & Aly, A. H. Simulation study of gas sensor using periodic phononic crystal tubes to detect hazardous greenhouse gases. *Sci. Rep.* **12**, 21553. 10.1038/s41598-022-26079-0 (2022). 36513778 10.1038/s41598-022-26079-0 PMC9747703

[26] Tammam, M. T. et al. Defected Photonic Crystal Array Using Porous GaN as Malaria Sensor, presented at the IOP Conference Series: Materials Science and Engineering. 10.1088/1757-899X/1171/1/012005 (2021).

[27] Zaky, Z. A., Panda, A., Pukhrambam, P. D. & Aly, A. H. The impact of magnetized cold plasma and its various properties in sensing applications. *Sci. Rep.* **12**, 3754. 10.1038/s41598-022-07461-4 (2022). 35260613 10.1038/s41598-022-07461-4 PMC8904597

[28] Zeng, C., Luo, C., Hao, L. & Xie, Y. The research on magnetic tunable characteristics of photonic crystal defect localized modes with a defect layer of nanoparticle. *Chin. Opt. Lett.* **12**, S11602. 10.3788/COL201412.S11602 (2014).

[29] Xiang, Y., Dai, X., Wen, S. & Fan, D. Enlargement of zero averaged refractive index gaps in the photonic heterostructures containing negative-index materials, *Phys. Rev. E* **76**, 056604. 10.1103/PhysRevE.76.056604 (2007). 10.1103/PhysRevE.76.056604 18233779

[30] Zaky, Z. A. & Aly, A. H. Novel smart window using photonic crystal for energy saving. *Sci. Rep.* **12**, 10104. 10.1038/s41598-022-14196-9 (2022). 35710799 10.1038/s41598-022-14196-9 PMC9203764

[31] Zaky, Z. A., Singh, M. R. & Aly, A. H. Tamm resonance excited by different metals and graphene. *Photonics Nanostruct. Fundam. Appl.* **49**, 100995. 10.1016/j.photonics.2022.100995 (2022).

[32] Zaky, Z. A., Amer, H. A., Suthar, B. & Aly, A. H. Gas sensing applications using magnetized cold plasma multilayers. *Opt. Quant. Electron.* **54**, 217. 10.1007/s11082-022-03594-y (2022).

[33] Zaky, Z. A., Al-Dossari, M., Zohny, E. I. & Aly, A. H. Refractive index sensor using fibonacci sequence of gyroidal graphene and porous silicon based on Tamm Plasmon Polariton. *Opt. Quant. Electron.* **55**, 6. 10.1007/s11082-022-04262-x (2023).

[34] Zaky, Z. A., Hanafy, H., Panda, A., Pukhrambam, P. D. & Aly, A. H. Design and analysis of gas sensor using tailorable fano resonance by coupling between tamm and defected mode resonance. *Plasmonics* **17**, 2103–2111. 10.1007/s11468-022-01699-4 (2022).

[35] Zaky, Z. A., Al-Dossari, M., Matar, Z. & Aly, A. H. Effect of geometrical and physical properties of cantor structure for gas sensing applications. *Synth. Met.* **291**, 117167. 10.1016/j.synthmet.2022.117167 (2022).

[36] Wang, Z. et al. 1D partially oxidized porous silicon photonic crystal reflector for mid-infrared application. *J. Phys. D* **40**, 4482. 10.1088/0022-3727/40/15/016 (2007).

[37] Ramirez-Gutierrez, C. F., Martinez-Hernandez, H. D., Lujan-Cabrera, I. A. & Rodriguez-García, M. E. Design, fabrication, and optical characterization of one-dimensional photonic crystals based on porous silicon assisted by in-situ photoacoustics. *Sci. Rep.* **9**, 1–15. 10.1038/s41598-019-51200-1 (2019). 10.1038/s41598-019-51200-1 PMC6791867 31611613

[38] Zhang, B. et al. Bandwidth tunable optical bandpass filter based on parity-time symmetry. *Micromachines* **13**, 89. 10.3390/mi13010089 (2022). 10.3390/mi13010089 PMC8780302 35056254

[39] Lova, P., Manfredi, G. & Comoretto, D. Advances in functional solution processed planar 1D photonic crystals. *Adv. Opt. Mater.* **6**, 1800730. 10.1002/adom.201800730 (2018).

[40] Zaky, Z. A., Sharma, A., Alamri, S. & Aly, A. H. Theoretical evaluation of the refractive index sensing capability using the coupling of Tamm–Fano resonance in one-dimensional photonic crystals. *Appl. Nanosci.* **11**, 2261–2270. 10.1007/s13204-021-01965-7 (2021).

[41] Ameen, A. A., Al-Dossari, M., Zaky, Z. A. & Aly, A. H. Studying the effect of quantum dots and parity-time symmetry on the magnification of topological edge state peak as a pressure sensor. *Synth. Met.* **292**, 117233. 10.1016/j.synthmet.2022.117233 (2023).

[42] Barvestani, J. & Mohammadpour, A. The effect of the centric graphene layer on the exceptional points of parity-time symmetric photonic crystals. *Opt. Quant. Electron.* **56**, 880. 10.1007/s11082-024-06754-4 (2024).

[43] Zaky, Z. A., Al-Dossari, M., Sharma, A. & Aly, A. H. Effective pressure sensor using the parity-time symmetric photonic crystal. *Phys. Scr.* **98**, 035522. 10.1088/1402-4896/acbcae (2023).

[44] Christodoulides, D. & Yang, J. *Parity-Time Symmetry and Its Applications* Vol 280 (Springer, 2018).

[45] Fang, Y., Zhang, Y. & Wang, J. J. Resonance-dependent extraordinary reflection and transmission in PC-symmetric layered structure. *Opt. Commun.* **407**, 255–261. 10.1016/j.optcom.2017.09.049 (2018).

[46] Klimov, V. et al. Optical gain and stimulated emission in nanocrystal quantum dots. *Science* **290**, 314–317. 10.1126/science.290.5490.314 (2000). 10.1126/science.290.5490.314 11030645

[47] Ma, G., Shen, J., Zhang, Z., Hua, Z. & Tang, S. H. Ultrafast all-optical switching in one-dimensional photonic crystal with two defects. *Opt. Express* **14**, 858–865. 10.1364/OPEX.14.000858 (2006). 19503405 10.1364/opex.14.000858

[48] Tsurumachi, N. et al. Enhancement of nonlinear optical effect in one-dimensional photonic crystal structures. *Jpn. J. Appl. Phys.* **38**, 6302. 10.1143/JJAP.38.6302 (1999).

[49] Ge, X. & He, S. Experimental realization of an open cavity. *Sci. Rep.* **4**, 1–5. 10.1038/srep05965 (2014). 10.1038/srep05965 PMC4122964 25096927

[50] Andueza, Á., Pérez-Conde, J. & Sevilla, J. Differential refractive index sensor based on photonic molecules and defect cavities. *Opt. Express* **24**, 18807–18816. 10.1364/OE.24.018807 (2016). 27505844 10.1364/OE.24.018807

[51] Panghal, A. et al. Terahertz chemical sensor based on the plasmonic hexagonal microstructured holes array in aluminum. In *44th International Conference on Infrared, Millimeter, and Terahertz Waves (IRMMW-THz)* 1–2. 10.1109/IRMMW-THz.2019.8874063 (2019).

[52] Sun, B., Yu, Y. & Yang, W. Enhanced toroidal localized spoof surface plasmons in homolateral double-split ring resonators. *Opt. Express* **28**, 16605–16615. 10.1364/OE.395068 (2020). 32549479 10.1364/OE.395068

[53] Aly, A. H. et al. Photonic crystal enhanced by metamaterial for measuring electric permittivity in GHz range. *Photonics* **8**, 416. 10.3390/photonics8100416 (2021).

[54] Qin, P. et al. Angle-insensitive toroidal metasurface for high-efficiency sensing. *IEEE Trans. Microwave Theory Tech.* **69**, 1511–1517. 10.1109/TMTT.2020.3027016 (2020).

