# Supplementary material for: Defected photonic crystal as propylene glycol THz sensor using parity-time symmetry
Source: Sci Rep. 2024 Oct 5;14:23209. doi: 10.1038/s41598-024-73477-7 (PMC11455864; doi:10.1038/s41598-024-73477-7)
Supplement: Supplementary file 1 — Supplementary Material 1 [file 41598_2024_73477_MOESM1_ESM.docx]

Defected photonic crystal as propylene glycol THz sensor using parity-time symmetry

Zaky A. Zaky ^1, 2, 3,^*, M. Al-Dossari ^4^, V. D. Zhaketov ^3,5^, and Arafa H. Aly ^1^

^1^ TH-PPM Group, Physics Department, Faculty of Science, Beni-Suef University, Beni Suef 62514, Egypt.

^2^ Academy of Scientific Research and Technology (ASRT), Cairo, Egypt

^3^ Frank Laboratory of Neutron Physics, Joint Institute for Nuclear Research, 141980, Dubna, Russia

^4^ Department of Physics, Faculty of Science, King Khalid University, Abha 62529, Saudi Arabia

^5^ Moscow Institute of Physics and Technology (State University), Dolgoprudnyi, Moscow oblast, Russia

***** Correspondence: zaky.a.zaky@science.bsu.edu.eg


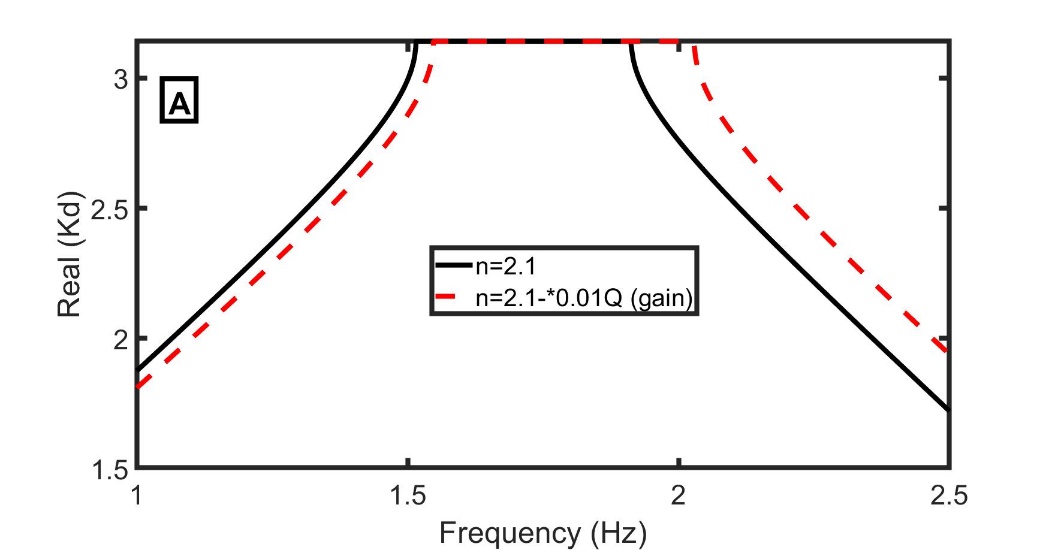


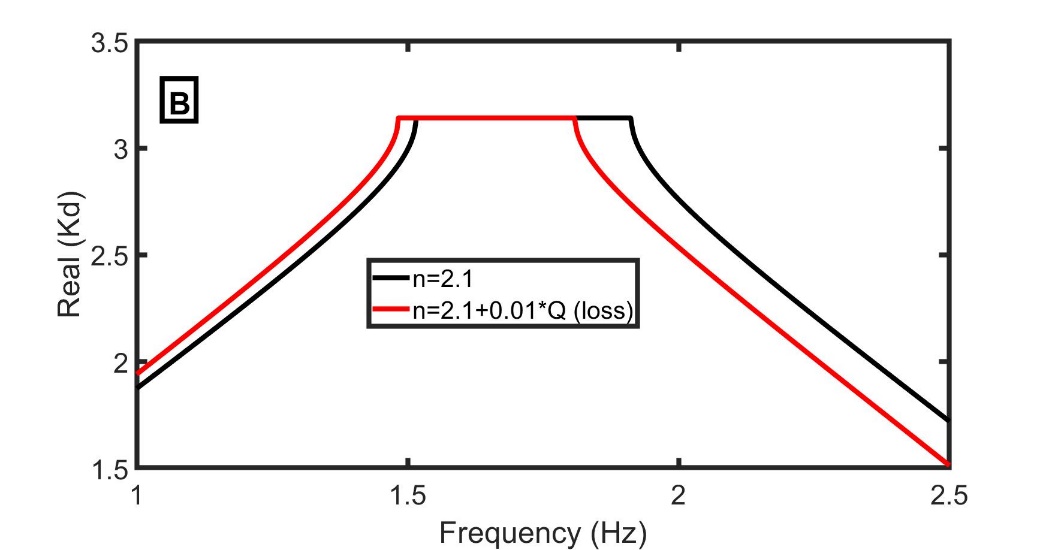


Sup. 1: The band structure for (A) the left photonic crystal (Gain/Si), and (B) the left photonic crystal (Loss/Si) at Q=12.4.
